# Canine testicular tumors: An 11-year retrospective study of 358 cases in Moscow Region, Russia

**DOI:** 10.14202/vetworld.2022.483-487

**Published:** 2022-02-26

**Authors:** Aleksey A. Gazin, Yury A. Vatnikov, Nikolay V. Sturov, Evgeny V. Kulikov, Viktor Grishin, Elena A. Krotova, Alisa A. Razumova (Varentsova), Natalia Yu. Rodionova (Sapego), Natalia I. Troshina, Varvara M. Byakhova, Ksenia V. Lisitskaya

**Affiliations:** 1 Veterinary Oncology Scientific Center, Veterinary Clinic “Biocontrol,” Moscow, Russia; 2 Department of Veterinary Medicine, Peoples’ Friendship University of Russia (RUDN University), Moscow, 117198, Russia; 3 Department of General Practice, Medical Institute, Peoples’ Friendship University of Russia (RUDN University), Moscow, 117198, Russia; 4 Department of Technosphere Safety, Peoples’ Friendship University of Russia (RUDN University), Moscow, 117198, Russia; 5 LABOKLIN RUSSIA, Moscow, Russia

**Keywords:** cryptorchid testes, interstitial cell tumors: mixed germ cell-sex cord-stromal tumors, seminomas, Sertoli cell tumors, testicular tumors

## Abstract

**Background and Aim:**

Canine testicular tumors are among the most common reproductive tract tumors in male dogs and have been studied in many countries. However, to the best of our knowledge, studies with a large sample size have not been conducted in Russia. This study aimed to provide the latest information on the prevalence of canine testicular tumors in the Veterinary Oncology Scientific Center for Small Animals “Biocontrol” in Moscow, Russia, in 2010-2020 and the characteristics of the affected canine population.

**Materials and Methods:**

A retrospective review of patients and histological reports was collected and analyzed from 358 dogs with 447 testicular tumors within 11 years.

**Results:**

The mean age of the affected dogs was 10.4 years, whereas that of dogs with Sertoli cell tumors was 9.4 years p=0.009. This study includes mixed-breed dogs (18.4%), Yorkshire Terriers (8.8%), Labrador Retrievers (7.9%), Golden Retrievers (5.0%), and Fox Terriers (3.4%). The most common tumors were interstitial cell tumors (n=227, 50.8%). In contrast, 107 (23.9%) seminomas, 80 (17.9%) Sertoli cell tumors, 19 (7.4%) mixed germ cell-sex cord-stromal tumors, and 26 (7.6%) testicular tumors developed from cryptorchid testes, which included 16 (61.5%) Sertoli cell tumors, 10 (38.5%) seminomas, and no interstitial cell tumors.

**Conclusion:**

This study provides baseline information on the prevalence of canine testicular tumors in the described population, including the median age of each tumor type and overrepresented dog breeds. We further found that the most common scrotal testicular tumor was interstitial cell tumor, whereas Sertoli cell tumor was the most common in cryptorchid testicles.

## Introduction

Testicles are the third most common anatomical site for tumor development in canines [1-4]. Testicular tumors, as the most common neoplasms in the male reproductive system in intact male dogs, account for up to 90% of cases [2,3,5]. Cryptorchidism and age are the main predisposing factors for the development of canine testicular tumors [2,6]. The mean age of dogs with testicular neoplasms is about 10 years [1,7-11]. Several studies have shown breed predisposition for testicular tumors, particularly in Sheltie, Collie, Boxer, German Shepherd, Fox Terriers, Afghan Hounds, and Norwegian Elkhounds [8,9,12]. However, the national prevalence of a breed must be considered.

Testicular tumors can arise from sex cord stroma (Sertoli and interstitial cell tumors), germ cells (seminoma, teratoma, etc.), or from both germ cells and sex cord stroma (mixed tumors) [5,13] but rarely from other histological structures (mesothelioma, rete testis epithelial tumors, etc.) [3,5,14-16]. The most common types of canine testicular tumors are interstitial cell tumors, seminoma, and Sertoli cell tumors; however, the incidence of these neoplasms varies among studies [7,9,10,12]. To the best of our knowledge, retrospective studies with a large sample size describing epidemiologic data on canine testicular tumors in Russia have not been published.

This study aimed to perform a retrospective study in dogs with testicular tumors and provide up-to-date information on the prevalence of different types of canine testicular tumors and the characteristics of the affected canine population in this area.

## Materials and Methods

### Ethical approval and Informed consents

The study was performed in accordance with the Russian Federation Legislation and the Guide for Research on Animals and was approved by the Bioethics Commission RUDN University (Moscow, Russia). Verbal consent was obtained from the owners of all the animals.

### Data source

A total of 15,446 histological cases were investigated at the private clinic Veterinary Oncology Scientific Center for Small Animals “Biocontrol” in Moscow, Russia during 2010 to 2020; 9826 cases of which involved dogs, 4949 of which were male. The cases included 391 surgical biopsy specimens of testicular tumors from 358 dogs presented for routine orchiectomy. All cases had partial or complete information, including records and slides. The records were reviewed for the following data: Age, breed, tumor type, and tumor localization. All information about patients was collected during pre-operative examinations with owner approval.

### Data preparation

Samples of canine testicular tumors were obtained after surgery and fixed in 10% neutral-buffered formalin for 24 h. Following fixation, the samples were dehydrated in alcohol (isopropyl) and embedded in paraffin. Morphologic features were evaluated on 4 μM sections stained with hematoxylin and eosin. Histological evaluation and tumor classification were performed using published criteria [5,13]. The tumors were classified into the most common canine testis tumors: Seminomas, Sertoli cell tumors, interstitial cell tumors, and mixed tumors (presence of more than 1 tumor type in one testicle). The classification criteria were growth patterns, characteristic features of each type of tumor (fibrovascular stroma, lymphocytic infiltration, and fibrosis), and cell morphology [5,13,14].

### Statistical analysis

One-way analysis of variance was performed to evaluate the relationship between age and tumor type. A 2×2 contingency table analysis and the Chi-square test were used to evaluate the relationship between canine testicular tumors and testicular localization (cryptorchid or non-cryptorchid). Statistical analysis was performed using the BioStat software (AnalystSoft Inc., California, United States), and the level of significance was p<0.05.

## Results

### Dataset

**Figure-1:**
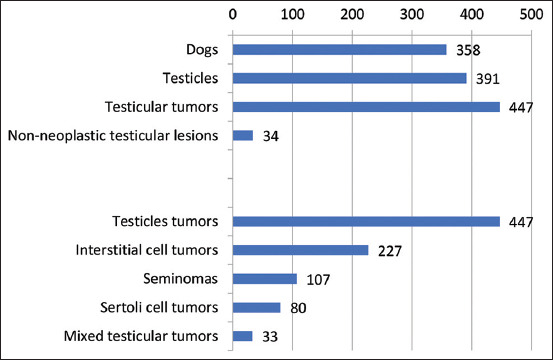
Results of the tumor classification in a representative population of dogs treated from 2010 to 2020. The total number of dogs participating in the study, the number of testes obtained from them, and neoplastic and non-neoplastic lesions. The results of the classification of the tumor lesions found are at the bottom of the graph.

From 2010 to 2020, 358 male dogs underwent orchiectomy with 391 testicles examined, 447 testicular tumors were histologically confirmed (Figure-1). In addition, 34 cases of non-tumor lesions were diagnosed, including orchitis, hemorrhage, ovotestis, and interstitial cell hyperplasia. Table-1 presents data characterizing the prevalence of dogs with testicular tumors in the populations presented in the study.

**Table 1:** Prevalence of dogs with testicular tumors at the Veterinary Oncology Scientific Center for Small Animals “Biocontrol” in Moscow, Russia.

Histopathological cases (2010-2020)	Number	% of canine testicular cases
Cats and dogs	15,448	2.32
Dogs	9,826	3.64
Male dogs	4,949	7.23

### Age distribution

**Figure-2:**
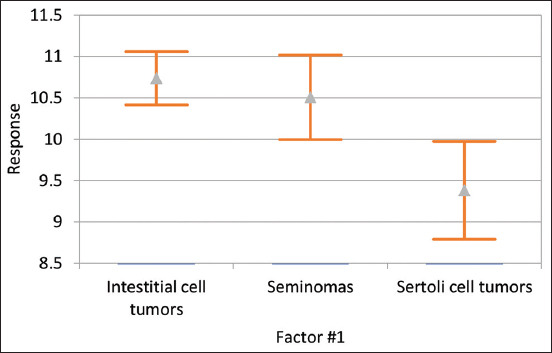
Analysis of variance of the relationship between age and tumor type. The results show that dogs with Sertoli cell tumors had a significantly lower mean age (9.4 years; p<0.009) than dogs with interstitial cell tumors (10.7 years) and seminomas (10.5 years) in our study.

The mean age of dogs diagnosed with testicular tumors was 10.4 years, with the youngest dog being only 2 years old and the oldest being 16 years old. The mean age differed with the tumor type: 10.5 years (range: 4-16 years) for seminomas, 10.7 years (range: 4-16 years) for interstitial cell tumors, and 9.4 years (range: 2-15 years) for Sertoli cell tumors. Dogs with Sertoli cell tumors had a significantly younger mean age (p<0.009) than those with interstitial cell tumors and seminomas (Figure-2).

### Breed information

**Figure-3:**
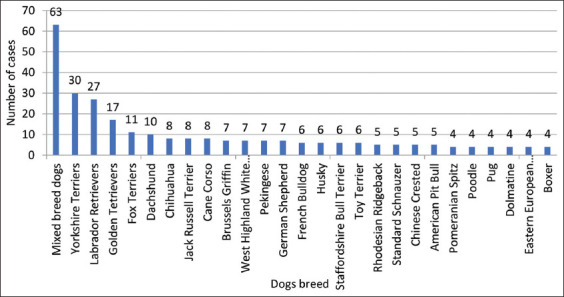
Number of cases of dog breeds represented. The graph shows all dog breeds with four or more testicular tumors. However, 34 dog breeds are not plotted because they had fewer than 4 testicular tumors.

Information regarding breed was obtained for 342 dogs. The following breeds were represented in the study: 18.4% (63/342) of mixed-breed dogs, 8.8% (30/342) of Yorkshire Terriers, 7.9% (27/342) of Labrador Retrievers, 5.0% (17/342) of Golden Retrievers, and 3.4% (11/342) of Fox Terriers (Figure-3).

### Testicular tumor information

A total of 447 canine testicular tumors were obtained from 358 dogs. According to histopathology results, 50.8% of cases (227/447) were diagnosed with interstitial cell tumors, 23.9% (107/447) of seminomas, 17.9% (80/447) of Sertoli cell tumors, and 7.4% (33/447) of mixed testicular tumors.

**Figure-4:**
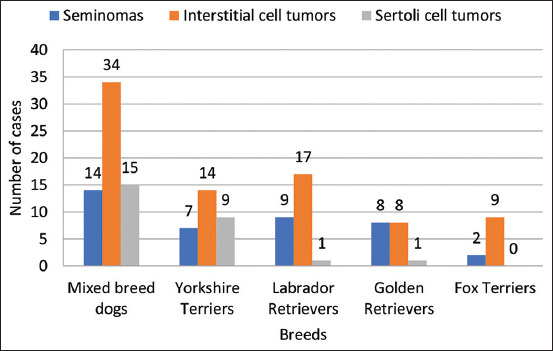
Most overrepresented canine breeds and their tumor types and number of cases. Interstitial cell tumors were predominant in mixed-breed dogs, Yorkshire Terrier, Labrador Retriever, and Fox Terrier. In our study, isolated cases of Sertoli cell tumors were reported in Labrador Retrievers and Golden Retrievers but none in Fox Terriers.

The breed was known in only 342 cases: 101 for seminomas, 172 for interstitial cell tumors, and 69 for Sertoli cell tumors. Except for mixed-breed dogs, seminomas were most common in Labrador Retrievers (9/101) and Golden Retrievers (8/101), interstitial cell tumors in Labrador Retriever (17/172) and Yorkshire Terriers (14/172), and Sertoli cell tumors, overwhelmingly, in Yorkshire Terriers (9/69) and Pekingese (5/69). More information about overrepresented breeds and the number of canine testicular tumors are presented in Figure-4.

### Testicular tumor location

Tumor localization was reviewed for 355 dogs. In total, 7.6% (26/355) of the tumors were localized in cryptorchid testes, 61.5% (16/26) of which were Sertoli cell tumors, and 38.5% (10/26) were seminomas. No cases of interstitial cell tumors were obtained from cryptorchid testes. Interstitial cell tumors are extremely rare in cryptorchid testicles than Sertoli cell tumors and seminomas; this was statistically significant (p<0.0001). The 2×2 contingency table analysis showed that 51% (104/204) of interstitial cell tumors, 49.4% (43/87) of seminomas, and 43.8% (28/64) of Sertoli cell tumors were found in the left testicle, whereas 56.2% (36/64) of Sertoli cell tumors, 50.6% (44/87) of seminomas, and 49.0% (100/204) of interstitial cell tumors were found in the right testicle. No significant difference was found between testicular tumor type and left or right testicle localization. The total number of each tumor type and tumor localization is summarized in Table-2.

**Table 2:** Canine testicular tumors and their localization.

Testicular tumors	Total	Left testis	Right testis	Cryptorchid testes
Interstitial cell tumors	227/447 (50.8%)	104/204 (51%)	100/204 (49%)	-
Seminomas	107/447 (23.9%)	43/87 (49.4%)	44/87 (50.6%)	10 (38.5 %)
Sertoli cell tumors	80/447 (17.9%)	28/64 (43.8%)	36/64 (56.2%)	16 (61.5%)

## Discussion

To the best of our knowledge, this study is the first large retrospective study and epidemiological analysis on testicular tumors in dogs in Russia. The average age of dogs with testicular tumors in our study was 10.4 years (range: 2-16 years). Our results are in accordance with those of the previous studies, in which seminomas, Sertoli cell tumors, and interstitial cell tumors are the most common types of canine testicular tumors [3,6,7,9,10,12,17,18].

The average age of dogs with testicular tumors in our study was 10.4 years (range: 2-16 years). This is quite similar to the findings of Liao *et al*. [9] and Manuali *et al*. [12]. To the best of our knowledge, there is no information on the presence of a significant difference in the mean age of dogs with different testicular tumors; however, our study showed that dogs with Sertoli tumors had a significantly lower mean age p=0.009 than those with interstitial cell tumors or seminomas.

Our study indicated that mixed-breed dogs (n=63) were overrepresented in our study, as well as Yorkshire Terriers (n=30) and Labrador Retrievers (n=27), but the national breed prevalence may limit these findings. A high number of Golden Retrievers (n=17) and Fox Terriers (n=11) were also noted in the study. Further research investigating a possible higher risk for the development of testicular tumors in these breeds is needed. These results can be used in clinical practice to determine the age groups at higher risk that should be more closely followed up, considering the average age of dogs with testicular tumors obtained in this study and considering the overrepresented and at-risk breeds [6,11,19].

A high incidence of seminomas was reported in several retrospective studies [7,9-12]; however, in our study, interstitial cell tumor was the most common testicular tumor type comprising up to 51.0% of all cases. The previous results still appear to be due to an overrepresentation of patients with cryptorchid testicles, whereas in our study, only 7.6% of tumors were localized in cryptorchid testes. Our study showed that seminomas were the second most common tumors in canine testicles, and these results are in accordance with data obtained by Grieco *et al*. [7], Manuali *et al*. [12], and Liao *et al*. [9], excluding findings on cryptorchid testicles.

Sertoli cell tumors were the most common type in cryptorchid canine testicles in the present study, which contrasts with the findings of Liao *et al*. [9]. This difference may be explained by the limited cases of cryptorchid testicular tumors reported. Further studies are required to establish the incidence of testicular tumors in canine cryptorchid testicles. In our study, interstitial cell tumors were not found in canine cryptorchid testicles, and they were rare in the studies by Liao *et al*. [9] and Nascimento *et al*. [10]. These results are likely related to the rarity of this tumor in canine cryptorchid testes.

No interstitial cell tumors were found in cryptorchid testes in our study, and other studies have reported that they were either rarely found or absent [9,10]. Thus, early castration of cryptorchid is recommended to prevent the development of Sertoli cell tumors and seminomas, which have greater potential for malignant behavior and a higher risk of metastasis than interstitial cell tumors [2,4,5,13,16].

The results obtained in this study on the prevalence of certain tumors, overrepresented breeds, and mean age for each tumor can help oncologists to correctly inform owners about the possible risks of developing tumors in intact dogs, including a significantly increased risk of seminomas and Sertoli cell tumors in cryptorchid testes, which agree with the data from other studies [6,9,18-20].

This study showed that seminomas, Sertoli cell tumors, and interstitial cell tumors were the most common types of canine testicular tumors in the Moscow Region of Russia. The incidence of these tumor types in canine testicles is similar to those reported in other retrospective studies [3,7,9,10,12]. However, in our study, Sertoli cell tumors were the most common type in cryptorchid testicles, whereas interstitial tumors were not found (p<0.0001). A significant difference between the mean age (p<0.009) of dogs with Sertoli cell tumors (9.4 years) and those with interstitial cell tumors (10.7 years) and Sertoli cell tumors (10.5 years) was identified. A high detection rate of testicular tumors was identified in mixed-breed dogs, Yorkshire Terriers, Labradors, Golden Retrievers, and Fox Terriers.

The study was limited by the lack of information on cryptorchidism in canines without testicular tumors during the study period. It did not identify the association between cryptorchidism and testicular tumor occurrence. Despite this limitation, other studies have demonstrated the association [9,11,12]. Second, the study did not evaluate the association between breed and testicular tumor occurrence due to the lack of information on canine breed populations in Moscow.

## Conclusion

Our retrospective study showed that testicular tumors are common in dogs studied in Moscow, Russia. The breed, age of the patient, and the location of the testes (cryptorchid or scrotal) can help veterinary oncologists determine the potential outcomes and risks of testicular tumors in dogs. The most common testicular tumor was interstitial cell tumors, except in cryptorchid testes, in which Sertoli cell tumors were the most common.

## Authors’ Contributions

AAG, KVL, YAV, and EVK: The original idea for the study and carried out the design. AAG, KVL, VMB, and EAK: Collected the data. AAG, NIT, NVS, VG, EVK, and AAV: Performed data analysis. AAG, YAV, NYS, NIT, and VMB: Drafted and revised the manuscript. All authors have read and approved the final manuscript.
